# Investigation of host–pathogen interaction between *Burkholderia pseudomallei* and autophagy-related protein LC3 using hydrophobic chromatography-based technique

**DOI:** 10.1186/s13578-017-0172-4

**Published:** 2017-08-23

**Authors:** Pattamaporn Joompa, Saranyoo Ponnikorn, Sittiruk Roytrakul, Sumalee Tungpradabkul

**Affiliations:** 1 Department of Biochemistry, Faculty of Science, Mahidol University, Bangkok, Thailand; 2 Chulabhorn International College of Medicine, Thammasat University, Rangsit Campus, Pathum Thani, Thailand; 3 National Center for Genetic Engineering and Biotechnology (BIOTEC), Thailand Science Park, Pathum Thani, Thailand

**Keywords:** *Burkholderia pseudomallei*, Autophagy, LC3, LC–MS/MS, Hydrophobic chromatography

## Abstract

**Background:**

*Burkholderia pseudomallei* is an intracellular bacteria causing Melioidosis, the disease widely disseminates in Southeast Asia and Northern Australia. *B. pseudomallei* has ability to invade various types of host cell and to interfere with host defense mechanisms, such as nitric oxide (NO). Due to the cross-talk among alternative killing mechanisms in host immune response against invading microbes, autophagy is the molecular mechanism belonging to intracellular elimination of eukaryotic cells that has been widely discussed. However, bacterial evasion strategy of *B. pseudomallei* and host-bacterial protein–protein interaction within autophagic machinery remain unknown.

**Methods:**

Here, we demonstrated the protein–protein interaction study between different strains of *B. pseudomallei*, including wild type PP844 and *rpoS* mutant, with autophagy-related protein LC3 that has been constructed, using the modified immunoaffinity hydrophobic chromatography based-technique. Liquid chromatography tandem-mass spectrometry (LC–MS/MS) analysis was utilized for identifying the eluted proteins obtained from the established column. In addition, the expression level of gene encoding candidate protein was predicted prior to verification using real-time quantitative reverse transcription PCR assay (RT-qPCR).

**Results:**

LC3 recombinant proteins could be entrapped inside the column before encountering their bacterial interacting partners. Based on affinity interaction, the binding capacity of LC3 with antibody displayed over 50% readily for hydrophobically binding with bacterial proteins. Following protein identification, bacterial ATP-binding cassette (ABC) transporter periplasmic substrate-binding protein (BPSL2203) was identified as a candidate LC3-interacting protein, which was found only in *B. pseudomallei* wild type. Gene expression analysis and bioinformatics of BPSL2203 were validated the proteomic result which are suggesting the role of RpoS-dependent gene regulation.

**Conclusions:**

Remarkably, utilization of the modified immunoaffinity hydrophobic chromatography with LC–MS/MS is a convenient and reliable approach to a study in *B. pseudomallei*-LC3 protein–protein interaction.

**Electronic supplementary material:**

The online version of this article (doi:10.1186/s13578-017-0172-4) contains supplementary material, which is available to authorized users.

## Background

*Burkholderia pseudomallei* is an intracellular gram negative bacteria causing melioidosis, a serious infectious disease, which has been reported in human and various animal species [1, 2]. The endemic areas of melioidosis are in Southeast Asia and Northern Australia. In Thailand, it is mostly encountered country, especially in the northeast of Thailand [2]. Currently, the outbreak of melioidosis has been reported that 165,000 cases were emerged among three billion people in endemic areas and displayed high rate of morbidity and mortality [3, 4]. The most clinical manifestation of melioidosis is septic shock that associated with bacterial dissemination to various organs [1]. Remarkably, *B. pseudomallei* has a unique intracellular lifestyle in various eukaryotic cells [5, 6]. After internalization in phagocytic cells, *B. pseudomallei* is able to escape from intracellular phagocytic and endocytic vacuoles, and followed by inducing actin polymerization to facilitate intracellular bacterial motility [7]. Furthermore, this pathogen can inhibit host innate immune response by interfering the inducible nitric oxide synthase (iNOS) expression in mouse macrophage cell line (RAW 264.7) [8], and can induce the multinucleated giant cells (MNGCs) formation for distributing bacterial infection to the adjacent cells before undergoing apoptotic cell death [9, 10]. Many virulence factors of *B. pseudomallei* have been characterized for the governing pathogenesis in host cells. RpoS sigma factor, one bacterial virulence factor has been studied the regulation in MNGCs formation and iNOS expression in both phagocytic and non-phagocytic cells [11, 12].

Since eukaryotic cells also promote their strategies to eliminate intracellular pathogens as same as induce the alternative cell death pathway, such as autophagy. Beside intracellular recycling pathway under the lysosomal-mediated degradative mechanism of cellular components, autophagy contributes to host immunity that conserved among eukaryotic cells to human [13, 14]. Many intracellular pathogens exhibit either evolved to evade autophagy-mediated killing or manipulated specific molecular mechanisms of autophagy for their survival inside the host cells, including *Shigella flexneri* [15], *Listeria monocytogenes* [16]. Among these bacterial species, type III secretion apparatus effecter proteins have been revealed the important roles in bacterial evasion from autophagy. In *S. flexneri,* IcsB, a type III secretion effector, masks a surface protein IcsA from the recognition of Atg5 protein underlying autophagy [15]. Whereas, a surface protein ActA plays a role in the recruitment of host actin-nucleation complex Arp2/3 to mask *L. monocytogenes* from the recognition of autophagy in host cytosol [16]. In *B. pseudomallei*, it has been studied the association with LC3 and the ability of bacterial avoidance in lysosomal-mediated killing process in mouse macrophage cell lines [17]. However, the manipulation of autophagy by *B. pseudomallei* as well as the interaction of bacterial proteins with autophagic proteins has been remaining unknown.

Host–pathogen interaction represents a complex and a dynamic biological system within microbial tactics and host defense mechanisms. Several innovative methods have been developed to identify and characterize protein–protein interaction between host and bacteria [18, 19]. To obtain the reliable results, various related techniques are required for verification [18]. The enrichment and isolation of protein–protein interaction have been studied in various types of molecular techniques, including immunoprecipitation, immunoaffinity technique, and tagged-labeling method [20, 21]. Recently high-throughput proteomic analysis has been applied for identification of relevant host–pathogen protein–protein interaction [22, 23]. Moreover, affinity chromatography combined with shotgun proteomics analysis has been practiced for investigating the interactions between *Streptococcus suis* proteins and host cells [24].

In this study, protein–protein interaction between LC3 and *B. pseudomallei* proteins has been identified and compared among wild type PP844 and *rpoS* mutant using the modified immunoaffinity hydrophobic chromatography coupled with liquid chromatography tandem mass spectrometry (LC–MS/MS). Interestingly, one protein belonging to bacterial ABC systems was detected as the LC3-interacting protein that found only in *B. pseudomallei* wild type but not *rpoS* mutant. Additionally, bioinformatic analysis and validation of BPSL2203 using RT-qPCR which are suggesting the role of RpoS-dependent gene regulation.

## Methods

### Cell culture

U937 cells (human monocytic cell line, ATCC CRL-1593.2) were cultured in RPMI 1640 medium (RPMI; Gibco Thermo Fisher Scientific, USA) supplemented with 10% heat-inactivated fetal bovine serum (FBS; Gibco Thermo Fisher Scientific, USA) and 100 unit/ml of penicillin–streptomycin (Gibco Thermo Fisher Scientific, USA). Cells were incubated at 37 °C in a humidified incubator with 5% CO_2_.

### Infection of *B. pseudomallei* in U937 cells

U937 cells were infected at an interested multiplicity of infection (MOI) following a previously established standard methodology [8]. Briefly, a total of 1 × 10^6^ cells was seeded into wells of a 6-well plate and incubated overnight under standard conditions. Cells were counted before co-culture with bacteria at MOI 20 and incubation for 24 h. Mock infection (no bacteria) was undertaken in parallel.

### Western blot analysis

U937 cells were co-cultured with bacteria under standard conditions following specified time point, then cells were washed with 1X PBS pH 7.4, and total proteins were extracted using RIPA buffer (Thermo Fisher Scientific, MA, USA). The expression levels of LC3-II and Glyceraldehyde-3-Phosphate Dehydrogenase (GAPDH) were determined by western blotting using a rabbit anti-LC3 polyclonal antibody (2775S; Cell Signaling Technology, MA, USA) and a mouse anti-GAPDH monoclonal antibody (611,463; BD Pharmacia, San Jose, CA, USA), and followed by a Horseradish peroxidase (HRP)-conjugated goat anti- rabbit IgG antibody (sc2004; Santa Cruz Biotechnology, Inc., TX, USA) and a HRP-conjugated goat anti-mouse IgG antibody (sc2005; Santa Cruz Biotechnology, Inc., TX, USA). The signal was visualized using Pierce^®^ ECL Western blotting substrate (Thermo Fisher Scientific, MA, USA).

### Indirect immunofluorescence assay

U937 cells from each experimental conditions were collected and counted approximately 5–10 × 10^4^ cells before removing the culture medium by centrifugation at 400×*g* for 5 min. The cells were washed once with RPMI and spun down onto glass cover slips using StatSpin^®^ CytoFuge 2 (Iris Sample Processing, USA) followed by the fixation method using ice-cold absolute methanol for 20 min before washing twice with PBS. Primary antibodies were a rabbit polyclonal anti-MAP- LC3 antibody (2775S; Cell Signaling Technology, MA, USA), a rabbit polyclonal anti-cathepsin D antibody (Ab-2, IM 16, Calbiochem; Merck KGaA, Darmstadt, Germany), and a mouse monoclonal anti-*Burkholderia pseudomallei* (Gifted from Dr. Narisara Chantratita, [25]). All primary antibodies were used at a concentration of 1:50. Secondary antibodies were Alexa™ 488 goat anti-mouse IgG antibody (Molecular Probes, Thermo Fisher Scientific, MA, USA) (1:200) and Alexa™ 594 goat anti-rabbit IgG antibody (Molecular Probes, Thermo Fisher Scientific, MA, USA) (1:200). Cells were examined using a laser scanning confocal microscope (LSM 510 Meta, Zeiss, Jena, Germany) with a 63× objective at zoom 2. The fluorescence intensity of 1000 cells from each experimental condition was determined using ImageJ 1.48v/Java software [Rasband WS: ImageJ US National Institutes of Health, Bethesda, Maryland, USA, 1997–2014. http://imagej.nih.gov/ij/. Accessed 1 Aug 2014]. The means of intensity from 1000 cells were presented as intensity ratios as colocalization, calculated from the intensity of *B. pseudomallei*-LC3 and *B. pseudomallei*-cathepsin D.

### Bacterial strains and protein extraction

*Burkholderia pseudomallei* PP844 and its *rpoS* mutant strains, which are isolated and constructed as previously described [8, 26], were cultured in the Luria–Bertani (LB) medium containing 100 μg/ml ampicillin at 37 °C until reaching the early stationary phase of growth (OD_600 nm_ 2.0–2.5). The bacteria was harvested and cell pellet was dissolved in PBS buffer pH 7.4 (137 mM NaCl, 2.7 mM KCl, 10 mM Na_2_HPO_4_, 2 mM KH_2_PO_4_) before adding 1% v/v of protease inhibitor cocktail set II (CalBiochem, La Jolla, CA). Bacterial protein extraction was done by sonication and separation at 10,000 rpm for 30 min at 4 °C. To determine protein concentration, Bradford method was utilized [27].

### Cloning and overexpression of *LC3* gene

*LC3* gene of Rattusnorvegicus (accession number: U05784) was amplified from mRFP-EGFP-LC3 vector [28]. Then, it was subcloned into the bacterial expression vector, pET17b. pET17b-LC3 was transformed into *Escherichia coli* BL21 (DE3). 1% inoculum of transformed bacteria was grown in LB medium for 3 h at 37 °C. 0.5 mM of isopropyl β-d-1-thiogalactopyranoside (IPTG) was added into the bacterial cultures and continuously incubated for 2 h at 37 °C. Bacterial protein was stored in PBS buffer pH7.4 supplied with 1% v/v of protease inhibitor cocktail set II prior to extraction by sonication.

### LC3-hydrophobic affinity column chromatography preparation

In this study, Albumin & IgG Depletion SpinTrap Column (GE Healthcare, USA), which is a conventional kit usable for hydrophobic column chromatography, was employed. The process of chromatography assay was operated according to the manufacturer’s protocol of kit. However, there was some modified steps in the protocol to support our strategy. Briefly, anti-LC3B antibody (Cell signaling technology, USA) at dilution 1:100 was applied to column and incubated at room temperature for 5 min. Whereas, 0.5 µg/µl LC3-expressed proteins and *B. pseudomallei* proteins were applied and leaved at room temperature for 1 h and 30 min, respectively. Finally, all bound proteins in column were eluted out using 0.1 M glycine–HCl, pH 2.7 before adding 1 M Tris, pH 9.5 to neutralize pH of elution system.

### Protein identification using LC–MS/MS

1.2 μg of total eluted proteins were firstly reduced using 20 mM DTT/10 mM NH_4_HCO_3_. Then, the mixtures were alkylated with 100 mM IAA/10 mM NH_4_HCO_3_. Denatured, reduced and alkylated proteins were digested using trypsin-to-protein at ratio of 1:20 (w/w) sequencing grade modified trypsin (Promega, Germany) and incubated at 37 °C overnight.

Tryptic digested peptides were protonated with 0.1% formic acid before individually injecting into NanoAcquity system (Waters Corp., Milford, MA, USA) equipped with a Symmetry C_18_ 5 μm, 180-μm × 20-mm Trap column and BEH130 C_18_ 1.7 μm, and 100-μm × 100-mm analytical reversed phase column (Waters Corp., Milford, MA, USA). The reference sprayer of the NanoLockSpray source of mass spectrometer was [Glu^1^] fibrinopeptide B. All tryptic peptides of all samples were analyzed using SYNAPT™ HDMS mass spectrometer (Waters Crop., Manchester, UK).

All mass spectra were determined and compared among different conditions of protein solution obtained from the columns using DeCyder MS 2.0 Differential Analysis software (GE healthcare, USA) [29]. PepDetect module automatically conducted to determine the mass spectra, assign the state of charge, and quantify protein concentrations based on MS signal intensities under MS mode. MS signal intensities among different conditions were subsequently aligned and compared to each other using PepMatch module. All MS/MS data were searched against MASCOT software (Matrix Science, London, UK) [30] and identified using National Center for Biotechnology Information (NCBI) *B. pseudomallei* database. Database search interrogation was accomplished as follows: enzyme (trypsin); fix modification (carbamidomethyl); variable modifications (oxidation of methionine residues); mass values (monoisotopic); protein mass (unrestricted); peptide mass tolerance (±2 Da); fragment mass tolerance (0.6 Da); peptide charge states (1+, 2+ and 3). Significantly different levels of proteins were analyzed using *t* test at *p* ≤ 0.05.

### Bioinformatics

All proteins were characterized the gene ontology analysis using UniProtKB (UniProt Knowledgebase) to identify biological process, molecular function, and cellular classification [31]. The mapping of protein–protein interaction to know the functional protein association networks was executed using STRING database [32]. The evidence mode was set up with a medium confidence level 0.4, and other search parameters were included: text mining, experiments, databases, co-expression, neighborhood, gene fusion, and co-occurrence. Protein–protein interaction network was analyzed from a collection of manually drawn pathway maps using KEGG (Kyoto Encyclopedia of Genes and Genomes) PATHWAY database [33].

### Promoter prediction

A training set of RpoS-dependent genes of *B. pseudomallei* has been created [34]. At the site 150 base pairs upstream region of gene encoding ABC transporter periplasmic substrate-binding protein of *B. pseudomallei* (*bpsl2203* gene) was predicted RpoS-dependent promoter by HMMER version 2.3.2, which is a Hidden Markov Model (HMM)-based program.

### Quantitative reverse transcription PCR (RT-qPCR)

Total RNA was isolated from *B. pseudomallei* PP844 and *rpoS* mutant under certain conditions using Trizol reagent (Invitrogen, USA). DNA contamination was tested before undergoing to the next steps. Then, cDNA synthesis was converted from 0.5 μg template RNA using ImProm II reverse transcriptase by following the manufacturer’s instruction (Promega, WI, USA). Specific primers were designed against *B. pseudomallei* K96243 annotated genome: ABCF-primer (5′-TTCGGATTCTCCACGATTCG-3′) and ABCR-primer (5′-GGACCGTCGTCATGTCGTAGTC-3′). The resulting cDNA was amplified by these primers and followed by mixing with the constituents of KAPA SYBR FAST qPCR kit (KAPA Biosystems, Inc., MA, USA) according to the manufacturer’ s protocol. Subsequently, two step real-time RT-PCR was performed using Stratagene Mx3005P (Agilent Technologies, CA, USA). Each samples of triplicate were identically prepared, and the means of the cycle threshold (C_T_) values were considered by program-defined threshold amount of fluorescence. *23* *s rRNA* gene was used as a reference gene for normalizing the difference of target gene expression. All results were analyzed the relative gene expression based on the Pfaffl method [35].

Statistical analysis was calculated from three independent experiments, each bacterial strains were carried out in triplicate. Using Sigma Plot 11.0 software, the Student’s paired *t*-test was used for evaluation of the significant differences, *p* values.

## Results

### Induction of autophagy in U937 infected with *B. pseudomallei*

**Fig. 1:**
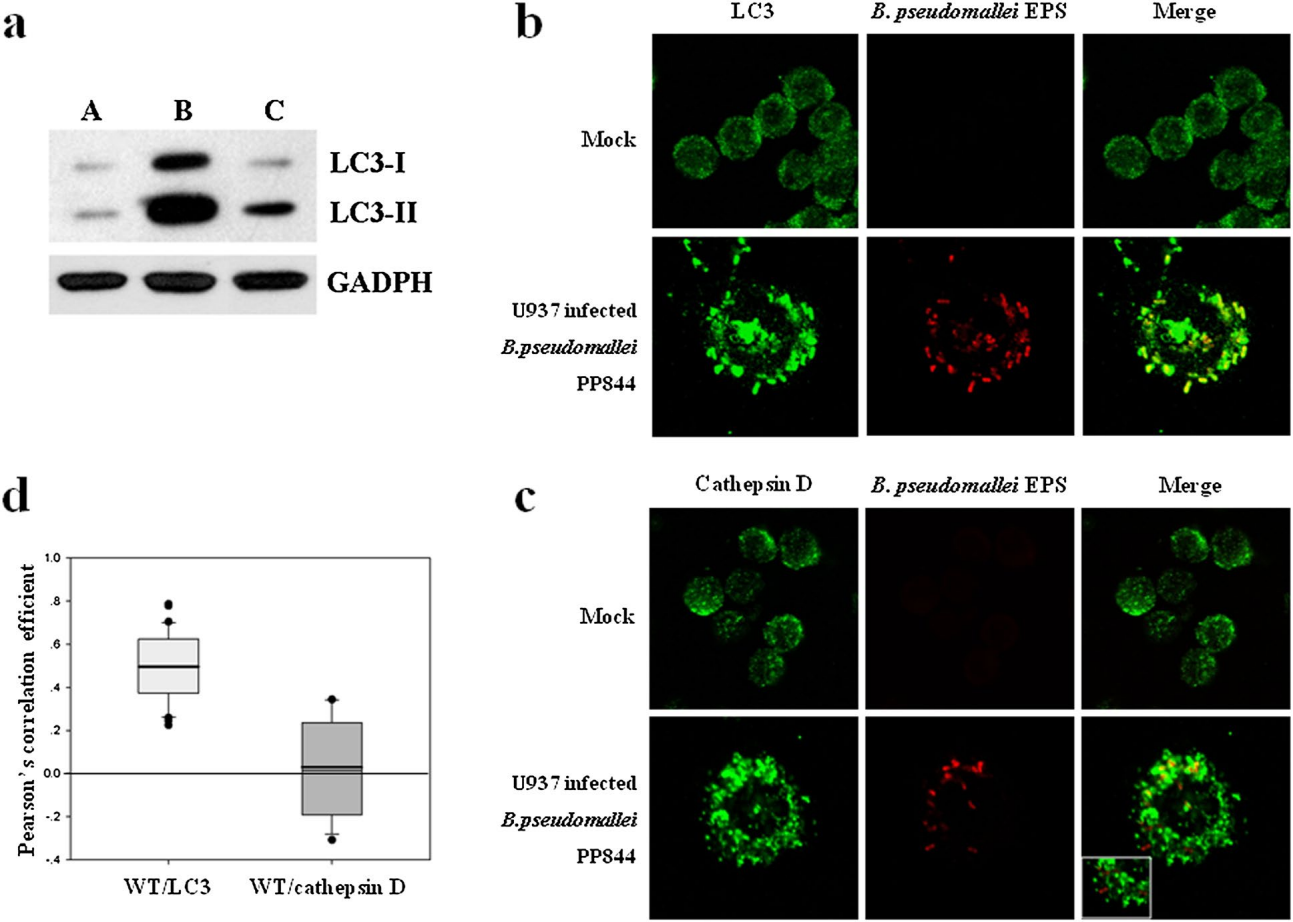
*Burkholderia pseudomallei* PP844 infection in U937 cell lines. **a** Western blot analysis verifying expression level of an autophagy-related protein LC3 in both isoforms; LC3-I and LC3-II, in U937 infected *B. pseudomallei* after 24 h of post infection at MOI20. Lane A represents to mock infection. Lane B and C represent to wild type PP844 and *rpoS* mutant infection, respectively. **b** Colocalization detection of *B. pseudomallei* (Alexa 488) and LC3 (Alexa 594). **c** Colocalization detection of *B. pseudomallei* (Alexa 488) and a lysosomal marker, cathepsin D (Alexa 594). **d** Pearson’s correlation coefficient (PCC) of colocalization from fluorescence intensity of bacteria with LC3 and bacteria with cathepsin D. A statistically significant difference between two processes of autophagy was determined using Mann–Whitney Rank Sum Test (*p* ≤ 0.001)

Since *B. pseudomallei* PP844 has been studied about the ability to suppress the expression of iNOS rather than *rpoS* mutant in both phagocytic and non-phagocytic cells [11, 12], the induction of an alternative host killing mechanism as autophagy in elimination of intracellular pathogen, *B. pseudomallei* has been addressed in murine macrophage [36]. To investigate the role of autophagy induced by *B. pseudomallei* infected human macrophage U937cell line, the expression level of microtubule-associated protein light chain 3 (LC3), a key autophagy-related protein was determined. Total extracted proteins were collected at 24 h of post infection time at MOI 20. The level of LC3-II was analyzed by western blotting, and GAPDH expression was determined as an internal control. The result showed the increase of LC3-II expression level in U937 infected *B. pseudomallei* wild type and *rpoS* mutant when compared to mock infection. Notably, LC3-II expression level of *rpoS* mutant was much lower than wild type (Fig. 1a). It means that *rpoS* mutant has less ability to induce autophagy in contrast with wild type. To further investigate the induction of autophagy from the infection experiment at 24 h post-infection and at MOI 20, immunofluorescence and confocal microscopy were utilized for observing the colocalization between LC3 and *B. pseudomallei* comparing with mock-infected condition. The result indicated the marked interaction of autophagy with bacteria during infection in U937 cells (Fig. 1b). The maturation of autophagy vacuoles as autophagolysosome fusion was identified by antibody against cathepsin D lysosomal marker and *B. pseudomallei*. The result showed that the colocalization of two markers was much diminished (Fig. 1c). Furthermore, colocalizations between *B. pseudomallei* with LC3 and *B. pseudomallei* with cathepsin D were estimated based on Pearson’s correlation coefficient (PCC) value, and were statistically tested by Mann–Whitney Rank Sum Test (*p* ≤ 0.001). The result revealed that a degree of colocalization between *B. pseudomallei* and LC3 is significantly higher than *B. pseudomallei* and cathepsin D (Fig. 1d).

### LC3 immunoaffinity hydrophobic column chromatography is an alternative mock model for protein–protein interaction study

Since antibody isotype IgG can be grabbed along with the prepacked column with protein G Sepharose [37], an appropriate concentration of both antibody and its specific protein was examined using the dot blot assay [38] prior to immunoaffinity hydrophobic chromatography assay. The results indicated that anti-LC3B antibody at dilution 1:100 was successfully entrapped inside the column (100% of binding capacity estimation) (Additional file 1: Figure S1). This modified immunoaffinity hydrophobic chromatography column with anti-LC3 antibody tagged bead was characterized the binding capacity prior to the next procedures. Firstly, the concentrations of LC3 recombinant protein at 4–0.25 µg/µl, could be applied to interact with this column (Additional file 1: Figure S1). At 0.5 µg/µl of recombinant protein was selected for binding with antibody-tagged bead. Approximately 75% of protein binding capacity was detected in the LC3-recombinant positive column. While, the percentage of protein binding capacity in column containing empty pET17b competent lysate binding with anti-LC3 antibody tagged bead was displayed less than 50% in negative control column (Table 1). Following modified immunoaffinity hydrophobic chromatography, the same concentration of LC3 recombinant protein was applied to column readily for encountering bacterial proteins. The result revealed that the binding capacity of bacterial protein in positive column of both strains were approximately 37% and 34% in wild type and *rpoS* mutant, respectively (Table 1). For elution process, the concentrations of eluted proteins in each experiment were determined, approximately 62% and 69% in wild type and *rpoS* mutant, respectively. However, the eluted protein concentration of negative control column was approximately 90%. Therefore, it was implied that this modified immunoaffinity hydrophobic chromatography column could be reliable to retain bacterial proteins as well as LC3 (Table 2). Finally, retained proteins inside each modified columns were eluted with 0.1 M glycine–HCl and calculated the percentage of protein elution as shown in Table 2. Here, these binding and eluting capabilities of proteins inside our modified immunoaffinity hydrophobic chromatography column are feasible and sufficient for further high throughput proteomic analysis.

**Table 1:** Comparative quantification of protein bound along column performing

Condition of column	Initial protein (µg ± SD)^a^	Flow-through (µg ± SD)^a^	Bound protein (µg ± SD)^a^	% of bound protein^b^
Column set up				
Recombinant LC3 binding control	50	12.67 ± 0.54	37.33 ± 0.54	74.65 ± 0.96
Empty pET17b competent binding control	50	25.58 ± 0.27	24.42 ± 0.27	49.24 ± 0.01
Bacterial protein (0.5 µg/µl) applying step				
*B. pseudomallei* WT	36.54 ± 0.54	23.08 ± 1.63	13.46 ± 1.09	36.87 ± 3.53
*B. pseudomallei rpoS* mutant	37.12 ± 1.36	24.42 ± 0.27	12.69 ± 1.63	34.14 ± 3.15
Negative control^c^	24.42 ± 0.27	19.42 ± 1.36	5.00 ± 1.09	20.50 ± 4.68

^a^Data was obtained from independent experimental replicate

^b^% of bound protein was calculated from bound protein (μg) × 100/initial protein existed in column (μg)

^c^Negative control represent to a column condition containing crude proteins without LC3 expression bait for protein of *B. pseudomallei* wild type (WT) strain

**Table 2:** Comparative quantification of eluted proteins obtained from each column condition

Condition of column	Bound protein (µg ± SD)^a^	Flow-through (µg ± SD)^a^	% of eluted protein^b^
Elution step using 0.1 M glycine–HCl pH 2.7			
* B. pseudomallei* WT	13.46 ± 1.09	8.30 ± 0.14	61.77 ± 2.89
* B. pseudomallei rpoS* mutant	12.69 ± 1.63	8.65 ± 0.11	68.56 ± 7.15
Negative control^c^	5.00 ± 1.09	3.95 ± 0.11	89.82 ± 13.26
Washing step			
* B. pseudomallei* WT	13.46 ± 1.09	4.60 ± 0.28	35.66 ± 2.10
* B. pseudomallei rpoS* mutant	12.69 ± 1.63	3.25 ± 0.18	26.59 ± 1.39
Negative control^c^	5.00 ± 1.09	ND	ND

^a^Data was obtained from independent experimental replicate

^b^% of eluted protein was calculated from eluted protein (μg) × 100/initial protein existed in column (μg)

^c^Negative control represent to a column condition containing crude proteins without LC3 expression bait for protein of *B. pseudomallei* WT strain

ND represent to no determination because protein concentration could not be detected based on Bradford assay

### Comparative bacterial proteins interacted with LC3 identification by the potential solution-based LC–MS/MS

LC3-interacting bacterial proteins in the modified immunoaffinity hydrophobic chromatography column from both *B. pseudomallei* wild type and *rpoS* mutant were eluted, digested in-solution, and followed by LC–MS/MS analysis. Total 237 proteins from all conditions were detected and annotated as the proteins of *B. pseudomallei*. MS signals of bacterial proteins of both wild type and *rpoS* mutant, which were similar to MS signals in negative control column, had been ruled out in this study. Decyder MS software module was used for comparative quantification in MS/MS intensity of LC3-interacting proteins among wild type and *rpoS* mutant. Nine annotated proteins with significant differences in relative level (*p* ≤ 0.05) were selected and intensively characterized using UniProtKB as described in Table 3. Of these, five annotated LC3-interacting proteins were found in both strains, including ATPase AAA (WP_004537907), Response regulator (ABN91906), and three hypothetical proteins (AFI66716, WP_011204911, ABA51435). However, three proteins, including type III secretion system ATPase (WP_011205607), hypothetical protein (WP_009927958), and partial Pca operon transcriptional activator PcaQ (WP_009948880), were detected only in *rpoS* mutant. Interestingly, ABC transporter periplasmic substrate-binding protein (WP_004535437) was only detected in wild type. This result postulated that RpoS sigma factor might regulate gene encoding ABC transporter periplasmic substrate-binding protein, which is a one possible bacterial protein candidate interacted with autophagy-related protein LC3.

**Table 3:** Protein identification compared between *B. pseudomallei* wild type (WT) and *rpoS* mutant in non-redundant sequence database (National Center for Biotechnology Information)

NCBI accession number	UniProt accession number	Protein ID	Function	ID score	WT intensity	*rpoS* mutant intensity
WP_004535437	A0A0E1ULU6_BURPE	ABC transporter periplasmic substrate-binding protein	Transporter activity	13.86	7.801	0
WP_011205607	Q63KG6_BURPS	EscN/YscN/HrcN family type III secretion system ATPase	ATP binding proton-transporting ATPase activity, rotational mechanism	8.19	0	10.503
WP_009927958	–	Hypothetical protein	–	0.02	0	8.970
WP_009948880	–	Pca operon transcription factor PcaQ, partial	–	16.54	0	9.642
WP_004537907	A8E9U6_BURPE	ATPase AAA	–	5.62	8.343	9.056
ABN91906	C4KQZ1_BURPE	Response regulator	DNA binding, phosphorylation signal transduction system	15.60	10.259	9.150
AFI66716	A0A0H3HLA2_BURP2	Hypothetical protein	–	12.39	9.870	6.858
WP_011204911	Q63WN9_BURPS	Hypothetical protein	–	7.60	9.240	10.529
ABA51435	Q3JLA7_BURP1	Hypothetical protein	–	7.31	8.264	9.84

To validate the identified peptides from LC–MS/MS analysis in *B. pseudomallei* genome, peptide sequence of ABC transporter periplasmic substrate-binding protein was analyzed. The query peptide sequences; REVPDGRFRAAAK, was aligned against the reference protein database using BLASTP (https://blast.ncbi.nlm.nih.gov/Blast.cgi).

The peptide sequence was matched to ABC transporter periplasmic substrate-binding protein of *B. pseudomallei* Parkistan9 (EEH25096). However, this strain has not been characterized the complete genome yet. Alternative peptide sequence alignment program was utilized to find and calculate the best-matching alignment between the query peptide sequence (EEH25096) and an annotated ABC transporter periplasmic substrate-binding protein of *B. pseudomallei* K96243, which has been already reported a complete genome [39]. The result showed that at amino acid position 1 to 441 of ABC transporter periplasmic substrate-binding protein of *B. pseudomallei* Parkistan9 was significantly matched with the template strain K96243 (E-value = 0) (data not shown). Therefore, ABC transporter substrate-binding protein (BPSL2203) of *B. pseudomallei* K96243 acts as an exponent of protein obtained from LC–MS/MS result.

**Fig. 2:**
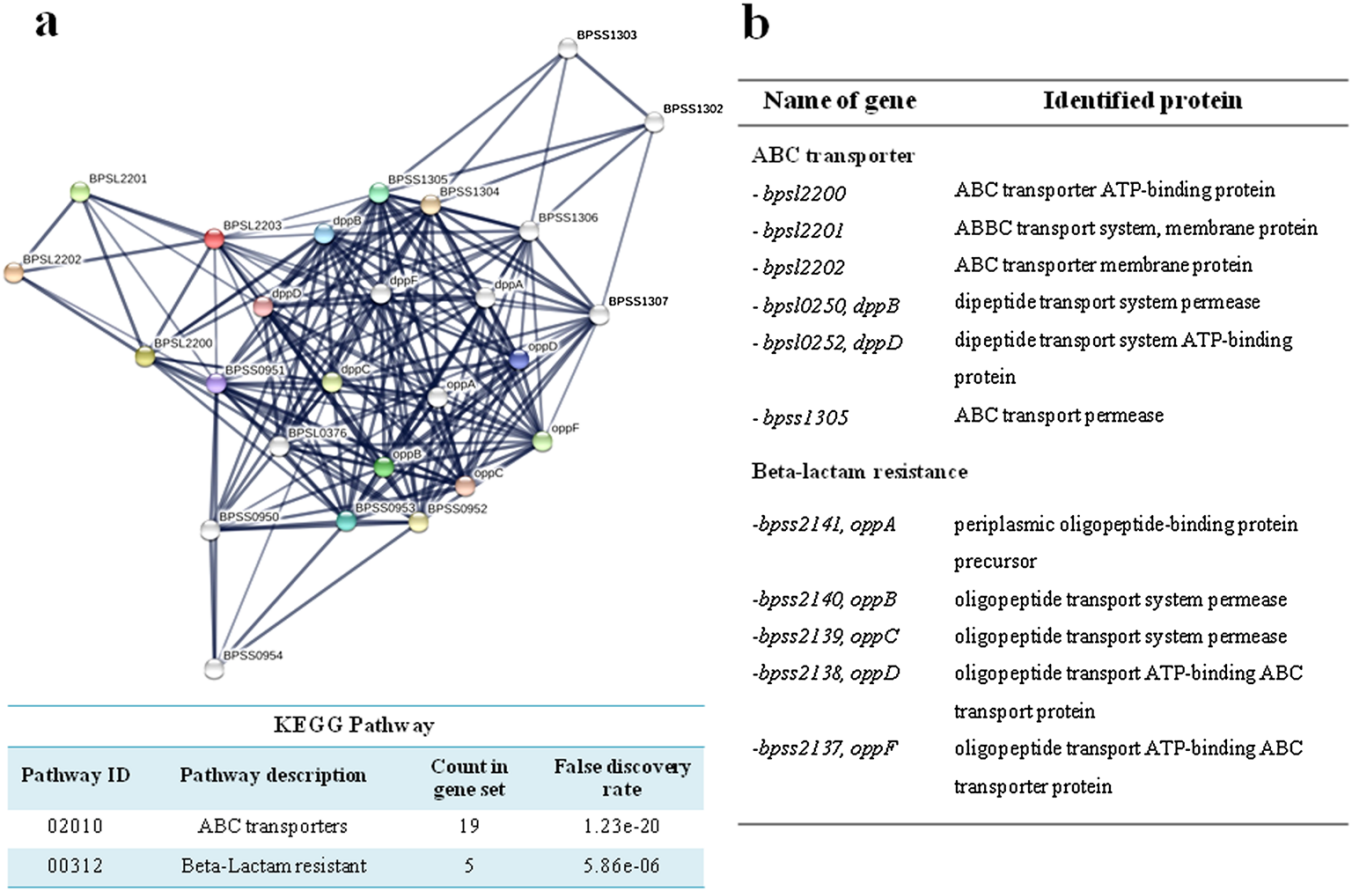
Protein–protein interaction network of ABC transporter periplasmic substrate-binding protein. **a** Protein-protein interaction neighborhood was illustrated by the confidence view of STRING 10.0 server. The group of neighborhoods was classified based on their biological processes and molecular functions using KEGG PATHWAY database. **b** Lists of selected proteins were revealed their involvement in biological processes, and descried their molecular functions

ABC transporter substrate-binding protein (BPSL2203) is defined its function as a substrate-binding protein component of oligopeptide transport system. The interaction of this candidate protein with the other functional proteins in *B. pseudomallei* was analyzed using STRING as shown in Fig. 2a. In addition, lists of functional partners were determined their characteristics in biological processes and molecular functions using KEGG PATHWAY database. Protein was considered as a significant candidate at a *p* value ≤ 0.05. The result revealed that BPSL2203 implicates with the ABC transporter pathway. It was particularly associated with the proteins encoded by its neighbor genes, including ABC transporter ATP-binding protein (BPSL2200), ABBC transport system, membrane protein (BPSL2201), ABC transporter membrane protein (BPSL2202), dipeptide transport system permease (BPSL0250), ABC transport permease (BPSS1305), dipeptide transport system ATP-binding protein (BPSL0252), and ABC transport permease (BPSS1305). Moreover, a group of gene *oppABCDF* encoding ATP-dependent oligopeptide transporter was represented the relevance with the beta-lactam resistance under KEEG database (Fig. 2b).

### RpoS sigma factor regulates ABC transporter periplasmic substrate-binding protein

**Fig. 3:**
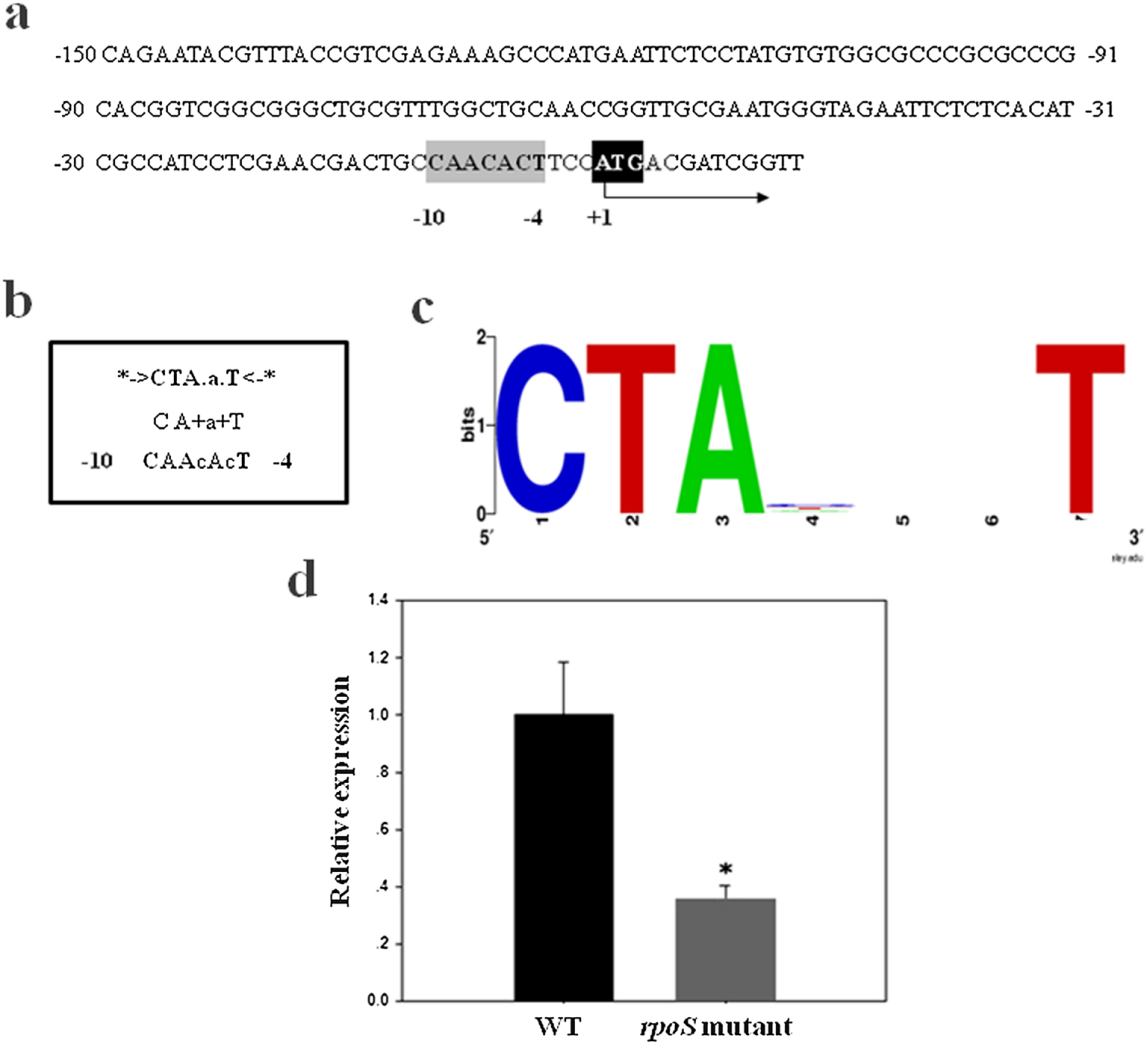
RpoS-dependent promoter prediction and quantification. **a** 150 nucleic acids upstream of complementary strand of gene encoding ABC transporter periplasmic substrate-binding protein (*bpsl2203*) was predicted RpoS-dependent promoter. **b** 10 promoter element was predicted by HMM analysis. The *first line* is representing to HMM consensus of RpoS promoter in *B. pseudomallei.* The *second line* is showing the letter perfectly matches to the consensus sequence of RpoS promoter, and the *third line* is the query sequence. **c** Sequence logo of RpoS-dependent promoter at −10 to −4 positions. **d** *bpsl2203* gene was relatively quantified comparing between wild type and *rpoS* mutant. Data were normalized by 23S rRNA expression level. *Asterisk* indicates to significant difference between these strains (*p* ≤ *0.01)*

From LC–MS/MS analysis, our results indicated that ABC transporter substrate-binding protein (BPSL2203) was only determined as a possible LC3-interacting protein in *B. pseudomallei* wild type. It postulated that RpoS could play an important role in regulating the expression of gene encoding candidate protein. RpoS-dependent promoter of gene encoding ABC transporter substrate-binding protein was predicted using HMM analysis. As expected to our hypothesis, promoter prediction result displayed that RpoS promoter locates at the position −10 to −4 upstream of *bpsl2203* gene (score = −7.7, E-value = 1) (Fig. 3a), which is greatly responding to a previous report of −10 region nucleotides of RpoS promoter [40]. Moreover, the effective RT-qPCR was utilized to validate RpoS-dependent regulation in ABC transporter substrate-binding protein (BPSL2203). Relative quantification result revealed that the expression of *bpsl2203* gene in *rpoS* mutant was significantly lower than wild type (*p* ≤ 0.01) (Fig. 3d). Regarding promoter prediction and RT-qPCR analysis, it was distinctly shown that the expression of gene encoding ABC transporter substrate-binding protein was under RpoS-dependent gene regulation. Likewise the result obtained from LC–MS/MS analysis, this LC3-interacting bacterial protein was detected only in *B. pseudomallei* wild type suggesting the role of RpoS during host–pathogen interaction underlying autophagy (Table 3).

## Discussion

Besides the maintaining homeostasis of eukaryotic cells, autophagy is one relevant immune mechanism to eliminate the pathogens in restricting bacterial replication and killing the intracellular pathogens under lysosomal degradation pathway [14]. Autophagy plays a critical role in accomplished elimination of invasion mechanism in some intracellular pathogens including, *Mycobacterium tuberculosis* [41], *Salmonella enterica* serovar Typhimurium [42], and Group A Streptococcus [43]. However, some bacteria can escape from autophagy including *Shigella flexneri* [15], *Listeria monocytogenes* [16], and also *Burkholderia pseudomallei* [17]. It has been shown that effector protein BopA, which is secreted via type III secretion system, is indicated to be the effector mediating autophagy evasion in *B. pseudomallei* [17]. However, fully immune-evasion strategies of *B. pseudomallei* have been under investigated. Here, we identify the induction of autophagy in phagocytic U937 cell line after *B. pseudomallei* wild type PP844 and *rpoS* mutant infections. The interaction of *B. pseudomallei* with autophagy-related protein LC3 has been obviously observed rather than bacteria with cathepsin D lysosomal marker. It has been concluded that *B. pseudomallei* is able to escape the killing mechanism under lysosomal enzyme function. On the other hand, *B. pseudomallei rpoS* mutant seems to lack the ability to trigger autophagy as shown as the expression level of LC3 in U937cells. Recent study, *B. pseudomallei rpoS* mutant has shown the low level of autophagy induction as well as the lower level of colocalization with LC3 in infected hepatocyte cell line, HC04 [12]. Interestingly, based on the guideline for the use and interpretation of autophagy [44], autophagic flux of *B. pseudomallei* wild type PP844 infected U937 comparing with *rpoS* mutant at 24 h of post infection time has already determined (Sanongkiet S, personal communication). After 24 h, *B. pseudomallei* wild type and *rpoS* mutant showed the increase levels of autophagic flux more than mock-infected condition. This supports our finding. RpoS, an alternative sigma factor implicates with the transcriptional regulation of genes in response to various stress environmental conditions and virulence genes involving host cell invasion [45]. This study could be confirmed that RpoS plays a critical role in host autophagy induction of both phagocytic and non-phagocytic cells. Due to the association of bacterial effector proteins with autophagy components, it has been recognized during intracellular bacterial infection. LC3 has been reported that it is recruited to the membrane around a small proportion of intracellular *S. flexneri*. In addition, colocalization of LC3 with type III secretion apparatus effecter proteins IpaB, IcsB, and VirA, was detected in the infected human colonic epithelial TC7 cells [46]. However, the understanding of host–pathogen interaction in *B. pseudomallei* has not been clarified yet.

To determine the interaction between LC3 and *B. pseudomallei* proteins, the modified immunoaffinity hydrophobic chromatography was developed in this study. Basically, protein–protein interaction studies are employed to virtually understand biological processes [18]. Nowadays, several methods have been utilized to approach and verify protein–protein interaction in both in vivo and in vitro models [18, 19]. Herein we try to generate a feasible and convenient method to approach protein–protein interaction study. Our strategy is performed based on the immunoaffinity technique between anti-LC3 antibody and LC3 recombinant protein within hydrophobic chromatography column. Hence, it is close to a typical method, co-immunoprecipitation (co-IP). Although co-IP is widely used for verifying protein–protein interaction, this technique requires high amount of protein samples and many steps of procedure should be concerned [47]. Using Albumin & IgG depletion spin trap column as a model for mimicking protein–protein interaction, a small amount of protein sample is required for applying into column as same as eluted protein is sufficiently provided for further exhaustively protein analysis. Moreover, the step of column spinning is meaningful for diminishing non-specific binding of unbound proteins or the other interferences. Whereas, the main protein clusters are still entrapped inside column (Table 1). Although polyclonal LC3B antibody was applied instead of monoclonal antibody; a properly used antibody in affinity purification, it is sufficient for selecting LC3 among crude extracted proteins of *E. coli* (data not shown). The result indicates that anti-LC3B antibody at dilution 1:100 is successfully entrapped inside the column (100% of binding capacity estimation) (Additional file 1: Figure S1). In this circumstance, percentage of binding capacity of LC3 recombinant protein has shown approximately 75% (Table 1), whereas binding capacities of bacterial proteins in the columns are approximately 35%. It might be explained in the context of hydrophobic interactions of proteins that are involved with several factors including, pH, temperature, type and concentration of the additive agents [48–50]. In addition, entrapped proteins were sufficiently eluted out of column for further analysis (Table 2).

Due to the complex and the consequent steps of autophagy regulation, lysosomal degradative pathway facilitates and cross-talks with many relevant immune functions for pathogen elimination, such as antigen processing, inflammation, and apoptosis [51]. Host–pathogen protein–protein interaction especially *B. pseudomallei* and autophagy has very few reports. Meanwhile, there is no evidence has been described the interaction of *B. pseudomallei* with LC3 whether it involves the pros and cons of bacterial pathogenesis. Our modified immunoaffinity hydrophobic chromatography column elucidates a potential strategy and less time-consuming for performing protein–protein interaction procedure. The study of LC3-interacting *B. pseudomallei* proteins was initiated based on the in vitro protein–protein interaction strategy coupled high throughput of protein identification from LC–MS/MS analysis [52]. Here, this is the first study of LC3-bacterial protein interaction. It is also verified a network inference with bioinformatics, predicted RpoS-dependent promoter, and quantified gene expression using RT-qPCR. These methods support and confirm the data obtained from LC–MS/MS suggesting ABC transporter periplasmic substrate-binding protein (*bpsl2203*) of *B. pseudomallei* wild type probably interacts with autophagy-related protein LC3 (Table 3). Interestingly, comparative study using our *B. pseudomallei rpoS* mutant [26], has been shown that ABC transporter periplasmic substrate-binding protein (*bpsl2203*) is under RpoS-dependent gene regulation. Therefore, this protein was not be detected in the mutant condition regarding LC–MS/MS analysis. Actually, peptide sequence matches to ABC transporter periplasmic substrate-binding protein of *B. pseudomallei* Parkistan9. Unfortunately, molecular function and association in biological processes of this gene encoding candidate protein in this strain have not been thoroughly described. The translated nucleotide of ABC transporter periplasmic substrate-binding protein of *B. pseudomallei* Parkistan9 is determined and found that it is close to an annotated *bpsl2203* gene of strain K96243. Based on NCBI database, *bpsl2203* is similar to gene encoding hypothetical protein precursor YejA, a periplasmic-binding subunit of an ABC transporter (YejABEF) complex in *E. coli*, which is function as a cargo receptor in part of peptide transporter. Using KEGG database, YejABEF complex has been defined that it belongs to the peptide and nickel ABC transporter family. Although biological function of YejABEF is not known, it has been reported the contribution with the resistance to antimicrobial peptides of *S. enterica* serovar Typhimurium and *B. melitensis* [53, 54]. Likewise, OppA, a periplasmic oligopeptide-binding protein precursor that belongs to the same family as YejABEF complex, has been previously shown the carrier-mediated transport of oligopeptides and contributed to intracellular survival of *L. monocytogenes* [55]. As our results showing in Figs. 2 and 3, RpoS has been reported to positively regulate ABC transporter genes in the transportation of oligopeptides (encoded by *oppABCDF*) in *E. coli* [56]. Previous study, ABC transporter encoded by *abcEDCBA* promoted the optimal expression of type IV secretion system in *Brucella ovis*. Proteins encoded *abcE*-*C* were identical with the group of ABC transporter uptaking dipeptides, oligopeptides, and nickel (Dpp/Opp/Nik). Interestingly, ABC transporter is necessary for intracellular evasion from phagolysosome fusion of this pathogen [57]. It could be implied this group of proteins in the peptide and nickel ABC transporter family that possibly synergize together for facilitating bacteria survival among the extreme environment inside host cells. Beside the implication of BPSL2203 in ABC transporter pathway based on STRING analysis, oppABCDF complex has been stated its involvement in the beta-lactam resistance (Fig. 2). The relevance of ABC transporter in bacterial resistance to beta lactam has been elucidated its contribution to various beta-lactam antibiotics in the innate resistance of *L. monocytogenes* [58]. Eventually, cellular mechanisms of RpoS-regulated ABC transporter of *B. pseudomallei* interacting autophagy are limited due to the lack of specific commercial antibody. However, this study should be further verified to reveal the functions of *B. pseudomallei* in cellular pathogenesis and host–pathogen interaction mechanism.

## Conclusions

Induction of autophagy in U937 infected with *B. pseudomallei* are associated with the increase of colocalization between bacteria and LC3. This is the first observation that was consequently initiated LC3-bacterial protein interaction study using the modified immunoaffinity hydrophobic chromatography column. Here, feasible and reliable technique for investigating protein–protein interaction demonstrated that ABC transporter periplasmic substrate-binding protein (BPSL2203) could be detected by LC–MS/MS analysis for its interaction with autophagy-related protein LC3. Moreover, using the consequent steps of validation could be concluded that the expression of gene encoding ABC transporter periplasmic substrate-binding protein is under RpoS-dependent gene regulation. However, the relevance of such protein of *B. pseudomallei* in bacterial manipulating host defense mechanism should be an important goal for further studies.

## Additional file

**Additional file 1: Figure S1.** Concentration optimization of anti-LC3 antibody and LC3 recombinant protein. (A) Anti-LC3 antibody was determined an appropriate concentration at dilution 1:20 and 1:100. Row 1 and 3, and row 2 and 4 represent to the amount of LC3 recombinant protein before and after applying into the column, respectively. Percentage of bound antibodies was estimated using ImageJ software program. (B) LC3 recombinant protein was investigated an appropriate concentration among 0.25 to 4 μg/μl.

## Abbreviations

LC3: microtubule-associated proteins 1A/1B light chain 3B
LC–MS/MS: liquid chromatography tandem mass spectrometry
NO: nitric oxide
iNOS: inducible nitric oxide synthase
