# Supplementary figures and images for: Investigation of host–pathogen interaction between Burkholderia pseudomallei and autophagy-related protein LC3 using hydrophobic chromatography-based technique
Source: Cell Biosci. 2017 Aug 23;7:45. doi: 10.1186/s13578-017-0172-4 (PMC5567900; doi:10.1186/s13578-017-0172-4)

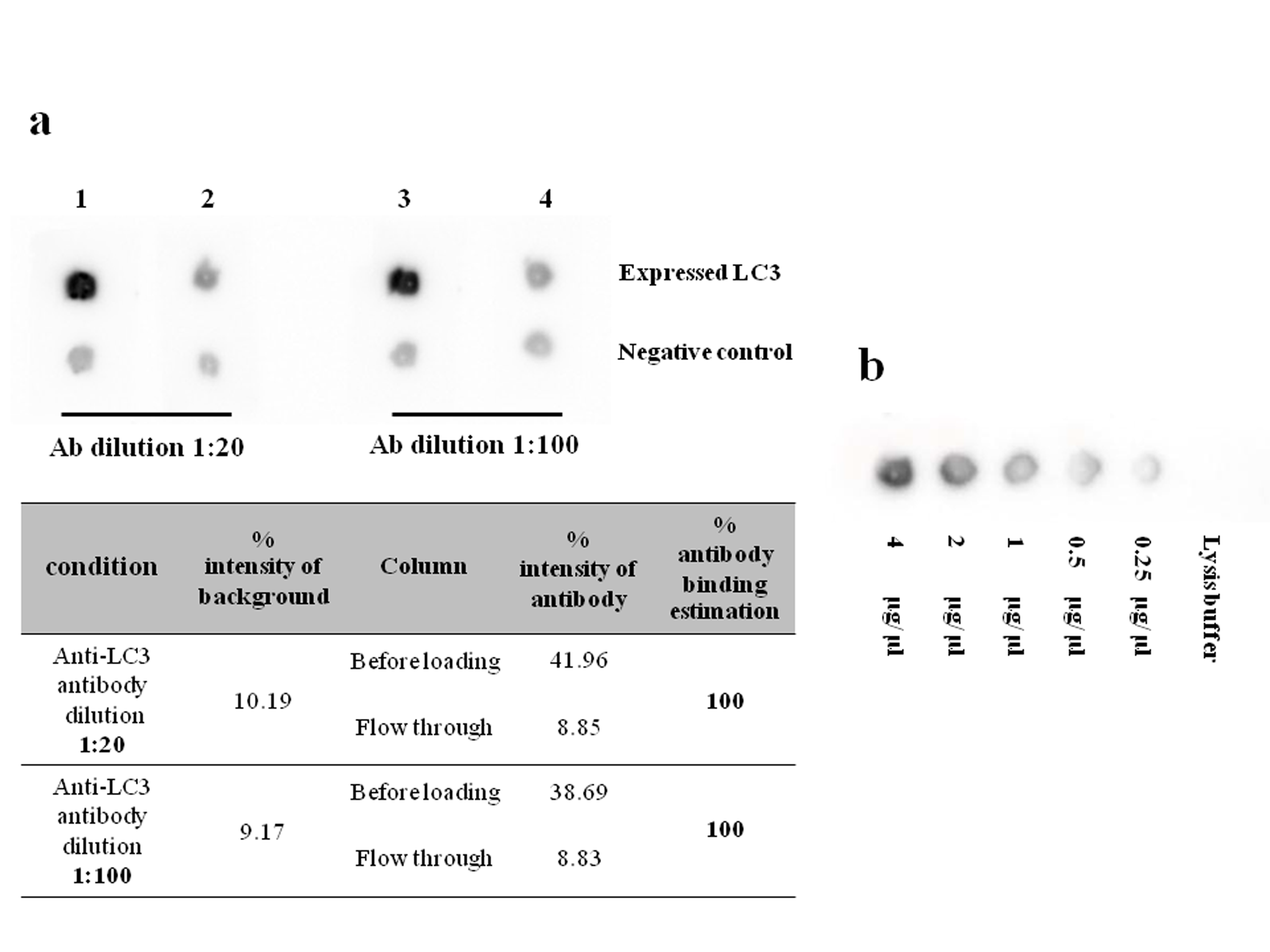

Supplement: Supplementary file 1 — Additional file 1: Figure S1. Concentration optimization of anti-LC3 antibody and LC3 recombinant protein. (A) Anti-LC3 antibody was determined an appropriate concentration at dilution 1:20 and 1:100. Row 1 and 3, and row 2 and 4 represent to the amount of LC3 recombinant protein before and after applying into the column, respectively. Percentage of bound antibodies was estimated using ImageJ software program. (B) LC3 recombinant protein was investigated an appropriate concentration among 0.25 to 4 μg/μl. [file 13578_2017_172_MOESM1_ESM.tif]
